# Case Report: Dual immunomodulatory and hematologic benefits of rituximab in refractory anemia of ANCA-associated vasculitis

**DOI:** 10.3389/fimmu.2025.1600250

**Published:** 2025-08-18

**Authors:** Ningjun Shao, Lingxiong Chai, Xu Bai, Qun Luo

**Affiliations:** Department of Nephrology, Ningbo No.2 Hospital, Ningbo, Zhejiang, China

**Keywords:** ANCA-associated vasculitis, rituximab, refractory anemia, hepcidin, case report

## Abstract

**Background:**

Antineutrophil cytoplasmic antibody (ANCA)-associated vasculitis (AAV) is a systemic autoimmune disease characterized by necrotizing small-vessel inflammation, frequently complicated by severe anemia and progressive renal injury. Anemia, affecting 73–92% of AAV patients, arises from multifactorial mechanisms including renal dysfunction, chronic inflammation, and iron dysregulation. Despite conventional immunosuppressive therapies, refractory anemia remains a significant challenge, with limited strategies targeting inflammation-driven hepcidin dysregulation.

**Case presentation:**

A 56-year-old woman presented with myeloperoxidase-specific antineutrophil cytoplasmic antibody (MPO-ANCA) positive AAV, transfusion-dependent anemia (hemoglobin: 56 g/L), and advanced chronic kidney disease with 55% tubulointerstitial atrophy. Initial management included cyclophosphamide, glucocorticoids, erythropoietin, and transfusions, yielding only a transient rise in hemoglobin (Hb) that rapidly declined despite treatment. Following the initiation of rituximab (RTX), her Hb level improved to 88 g/L within four weeks and normalized to 127 g/L after four biweekly infusions (500 mg each). Concurrently, MPO-ANCA titers decreased from 1:1280 to 1:80, and pulmonary infiltrates resolved. However, renal function remained impaired (serum creatinine: 229 µmol/L) due to irreversible fibrosis.

**Conclusions:**

This case demonstrates RTX’s dual efficacy in suppressing autoimmunity and alleviating anemia, potentially through indirect effects on inflammatory pathways and iron metabolism. Early RTX use may reduce transfusion dependency and help stabilize renal function in refractory AAV, though advanced fibrosis limits recovery. These findings support RTX as a first-line option in AAV patients with severe anemia and evolving renal injury.

## 1 Introduction

Antineutrophil cytoplasmic antibody (ANCA) -associated vasculitis (AAV) is a systemic autoimmune disorder characterized by necrotizing inflammation of small vessels, frequently complicated by severe anemia and progressive renal injury (1). Anemia, observed in 73–92% of AAV patients at diagnosis, arises from multifactorial mechanisms including renal erythropoietin deficiency, chronic inflammation, alveolar hemorrhage, malnutrition, iron deficiency and occult gastrointestinal bleeding (2, 3). Notably, anemia severity correlates with tubulointerstitial damage and predicts mortality, yet targeted therapeutic strategies remain limited. While rituximab (RTX), a CD20^+^ B-cell depleting agent, is established for refractory or relapsing AAV, its broader immunomodulatory impact, including potential benefits on hematologic parameters, remains incompletely understood (4).

Here, we present the case of a patient with myeloperoxidase-specific antineutrophil cytoplasmic antibody (MPO-ANCA) positive AAV who suffered from severe, transfusion-dependent anemia and advanced tubulointerstitial atrophy that was unresponsive to standard therapy. After initiating RTX, the patient’s hemoglobin (Hb) levels normalized without further transfusions, coinciding with a reduction in MPO-ANCA titers. This case highlights the dual capacity of RTX to suppress pathogenic autoimmunity while also alleviating inflammation-associated anemia, suggesting its potential as a therapeutic strategy for patients who are refractory to traditional immunosuppressants.

## 2 Case presentation

A 56-year-old woman presented to the emergency department with a one-month history of progressive fatigue and dizziness on exertion. She reported black stools over the preceding week, suggestive of occult gastrointestinal bleeding, but denied fever, chills, or acute abdominal pain. Her condition worsened acutely one day prior to admission, marked by severe generalized weakness, nausea with one episode of non-bloody vomiting, palpitations, and cold sweats. Initial laboratory evaluation revealed profound anemia (hemoglobin: 56 g/L) and acute kidney injury (serum creatinine: 255.3 µmol/L), prompting urgent hospitalization.

On admission, physical examination revealed an alert but fatigued patient with marked pallor. Her heart rate was 103 bpm and blood pressure was 149/93 mmHg. No jaundice, lymphadenopathy, or cutaneous hemorrhages were observed. Cardiopulmonary auscultation demonstrated regular heart sounds without murmurs and bilateral fine crackles at the lung bases. The abdominal examination was unremarkable. Neurological assessment was normal.

**Figure 1:**
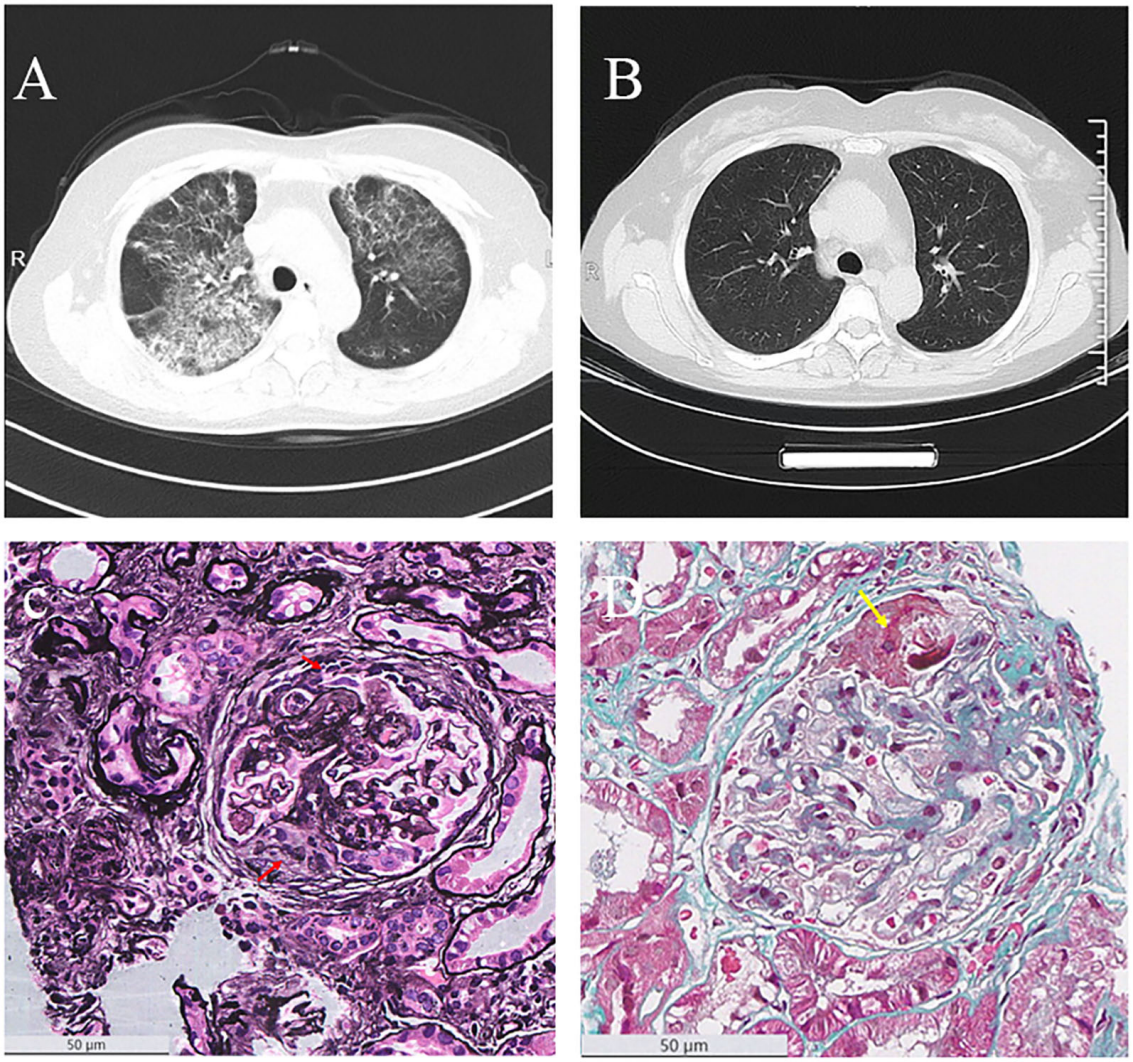
Radiological and pathological findings. **(A)** Chest CT image before treatment, showing pulmonary lesions. **(B)** Chest CT image after four doses of rituximab (RTX) therapy, demonstrating improvement in pulmonary lesions. **(C)** PASM staining of kidney biopsy (×400), with red arrows indicating cellular fibrocellular crescents. **(D)** MASSON staining of kidney biopsy (×400), with yellow arrows indicating fibrinoid necrosis.

Chest computed tomography (CT) revealed diffuse bilateral ground-glass opacities and patchy consolidations, suggestive of interstitial pneumonia. There was no evidence of cardiogenic pulmonary edema or diffuse alveolar hemorrhage (**Figure 1**). Elevated inflammatory markers, including C-reactive protein (CRP) at 85.73 mg/L, supported systemic inflammation. Serological testing confirmed MPO-ANCA positivity (titer: 1:1280). Additional laboratory findings included elevated ferritin (271.6 ng/mL) and hypoalbuminemia (25.4 g/L). Urine testing on admission showed significant proteinuria, with a urine albumin-to-creatinine ratio (ACR) of 3289 mg/g. A renal biopsy demonstrated pauci-immune necrotizing glomerulonephritis with chronic vascular injury. Of the 42 glomeruli available for examination, 28 were globally sclerotic and 5 were segmentally sclerotic. The biopsy also showed non-active chronic lesions, including fibrocellular crescents and tubulointerstitial fibrosis involving 55% of the parenchyma (**Figure 1**). These findings confirmed chronic-phase renal injury consistent with AAV.

Initial management aimed to address the severe anemia and active vasculitis. The patient received three blood transfusions (total 5 units) alongside recombinant human erythropoietin (rHuEPO) (10,000 IU weekly) and oral iron supplementation. Immunosuppression was initiated with methylprednisolone (40 mg twice daily for three days), followed by 40 mg once daily. One dose of cyclophosphamide (CTX, 0.6 g IV) was administered; however, given the lack of clinical improvement and ongoing transfusion dependency, further doses were withheld. Despite a transient post-transfusion rise in hemoglobin (peak: 87 g/L), levels rapidly declined to below 60 g/L. Fecal occult blood testing was weakly positive; however, both gastroscopy and colonoscopy ruled out active gastrointestinal bleeding. A comprehensive anemia workup was performed to exclude other causes (**Supplementary Table 1**). Hemolysis was ruled out by a negative direct Coombs test and normal levels of lactate dehydrogenase, total, and indirect bilirubin. Vitamin B12 and folate levels were within normal ranges, excluding nutritional deficiencies. Although serum ferritin was elevated (271.6 ng/mL) and transferrin saturation exceeded 15%, these findings were consistent with functional iron restriction rather than absolute iron deficiency. These findings supported a diagnosis of anemia of chronic disease (ACD).

In light of persistent vasculitis activity, manifested by pulmonary infiltrates and elevated MPO-ANCA titers, and refractory anemia unresponsive to prior therapy, RTX (500 mg every two weeks) was initiated after ruling out contraindications.

**Figure 2:**
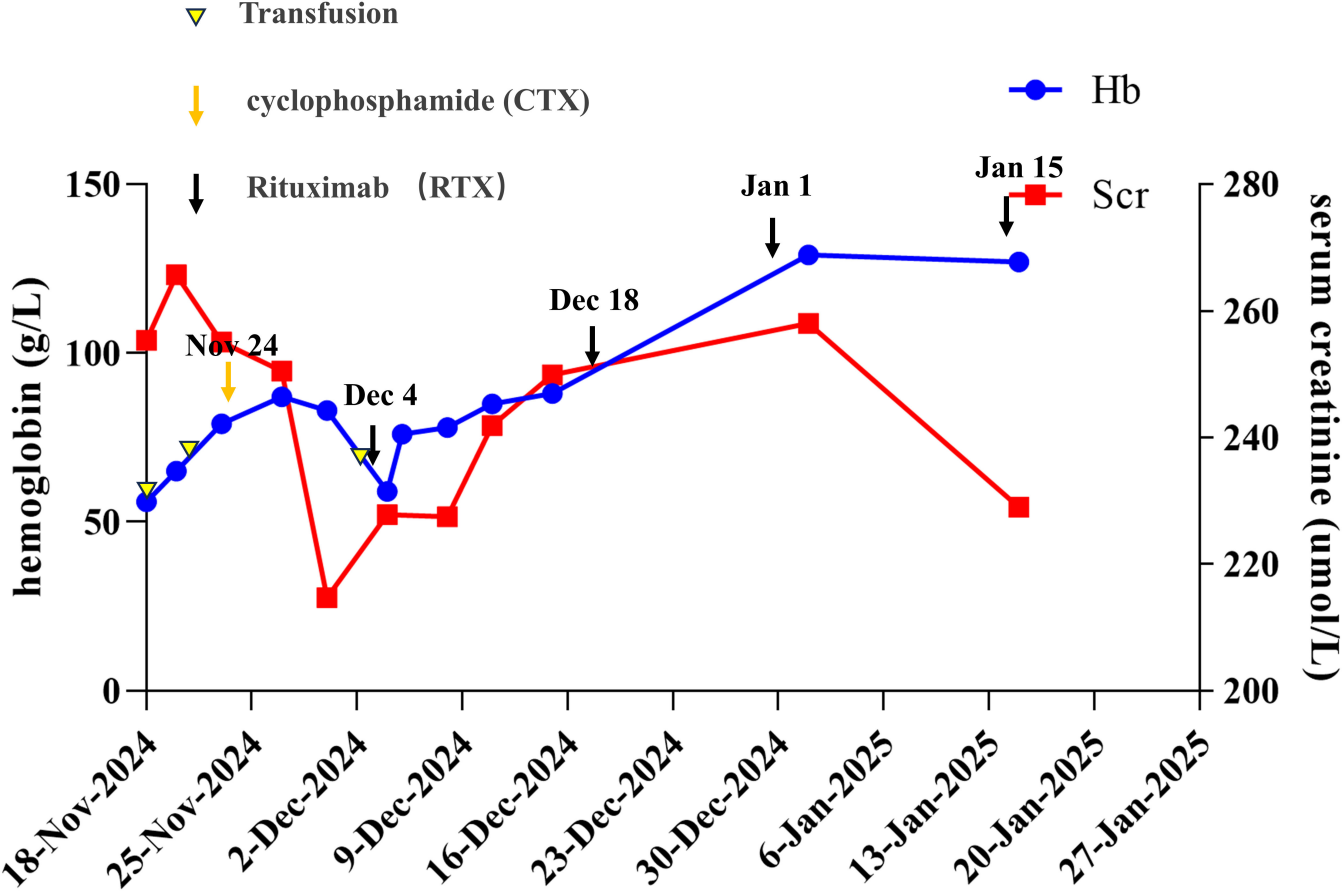
Overview of the patient’s clinical course and management. The timeline illustrates the patient’s clinical progression, including changes in hemoglobin levels, serum creatinine levels, and the timing of rituximab (RTX) therapy and transfusions. Key clinical events and treatment responses are documented from November 18, 2024, to January 27, 2025.

Follow-up at four weeks post-RTX initiation demonstrated hematologic improvement (hemoglobin: 88 g/L), with normalization to 127 g/L achieved after four RTX infusions (total cumulative dose: 2,000 mg). This fixed-dose regimen was chosen based on recent evidence showing that a 500 mg biweekly schedule offers similar efficacy to the standard 375 mg/m² weekly protocol for AAV, especially in patients with renal involvement, while being more convenient (5, 6). Repeat MPO-ANCA testing showed a reduced titer (1:80), and ferritin levels decreased to 156 ng/mL. A repeat chest CT revealed resolution of the pulmonary infiltrates. Urinary protein also improved, with the urine ACR decreasing to 2120.5 mg/g and a 24-hour urine protein excretion of 2470.4 mg/24h. However, chronic kidney disease persisted (serum creatinine: 229 µmol/L), reflecting the irreversible fibrosis observed on biopsy. No disease flares occurred during a two-month monitoring period. The clinical course is summarized in **Figure 2**.

## 3 Discussion and conclusion

The profound anemia in this patient illustrates the complex pathophysiology of anemia in AAV. While severe renal insufficiency (estimated glomerular filtration rate (eGFR) <30 mL/min/1.73m²) likely contributed to erythropoietin deficiency, the lack of a sustained hemoglobin response to transfusions and rHuEPO suggested a dominant role for inflammation-driven ACD (7). Elevated ferritin and CRP are hallmark features of ACD, driven by pro-inflammatory cytokines, particularly interleukin-6 (IL-6), which induces hepatic hepcidin production (8). Hepcidin binds to ferroportin on macrophages and enterocytes, blocking iron efflux and sequestering iron intracellularly, thereby impairing iron availability for erythropoiesis (9). This results in a functional iron deficiency: erythropoiesis is impaired due to restricted circulating iron despite adequate total body iron stores.

In this case, no overt infection was identified, but the chronic inflammatory state inherent to AAV likely triggered the same hepcidin-mediated iron restriction mechanisms seen in infection-related ACD (10, 11). The combination of elevated ferritin (>270 ng/mL), high CRP (>85 mg/L), and a transferrin saturation >15% supported the diagnosis of ACD over pure iron deficiency anemia (12). Additionally, the exclusion of hemolysis and active gastrointestinal bleeding reinforced inflammation as the dominant contributor to her anemia.

The patient’s clinical course improved markedly after initiating RTX. As a CD20-targeted monoclonal antibody, RTX depletes autoreactive B cells responsible for ANCA production, thereby attenuating neutrophil activation, endothelial injury, and the IL-6-driven inflammatory cascade (13). The observed decrease in MPO-ANCA titers and CRP levels paralleled the improvement in hemoglobin. This suggests that RTX may have indirectly restored erythropoiesis by modulating inflammatory pathways that drive hepcidin production (14, 15), although serum hepcidin was not measured in this case. RTX may also alleviate anemia through broader immunomodulatory effects, including the downregulation of other proinflammatory cytokines such as TNF-α, which can suppress marrow inhibitory signaling and restore erythroid precursor proliferation (16, 17).

In contrast to CTX, which has broad cytotoxic effects, RTX offers a more targeted mechanism. Our patient’s anemia persisted during CTX treatment but responded promptly to RTX. CTX reduces inflammation through generalized immunosuppression, but its effect on the IL-6 and hepcidin pathways central to ACD is less direct (18, 19). By depleting B cells, RTX more specifically attenuates the IL-6-driven inflammatory cascade (13, 20), which likely relieved the inflammation-induced block on iron metabolism and contributed to the hematologic recovery observed (21).

The limited renal recovery in this patient demonstrates the long-term consequences of delayed intervention. The renal biopsy revealed extensive irreversible damage, including 55% tubulointerstitial fibrosis and a high percentage of globally sclerotic glomeruli. While RTX successfully halted further immunologic injury, it could not reverse chronic scarring. The patient’s persistent elevation in serum creatinine and only partial improvement in proteinuria (ACR decreased from 3289 to 2120.5 mg/g) reflect this residual structural damage. These findings reinforce the concept of a “therapeutic window,” in which early initiation of RTX, prior to the onset of irreversible fibrosis, is crucial to preserving long-term renal function (22).

In conclusion, this case demonstrates that RTX can effectively resolve severe, inflammation-driven anemia in AAV, even when significant renal fibrosis limits organ recovery. The patient’s transition from transfusion dependency to sustained hematologic remission suggests that RTX helped overcome functional iron restriction by suppressing the underlying inflammatory drivers of ACD. Although mechanistic biomarkers like serum hepcidin were not measured, the clinical response supports the hypothesis that RTX acts by modulating immune-mediated iron dysregulation. These findings suggest that for AAV patients presenting with severe anemia and active inflammation, early consideration of RTX may be warranted to reduce transfusion requirements and improve hematologic outcomes, regardless of the potential for renal function recovery.

## Data Availability

The original contributions presented in the study are included in the article/**Supplementary Material**. Further inquiries can be directed to the corresponding author.
