# Supplementary material for: Case Report: Dual immunomodulatory and hematologic benefits of rituximab in refractory anemia of ANCA-associated vasculitis
Source: Front Immunol. 2025 Aug 18;16:1600250. doi: 10.3389/fimmu.2025.1600250 (PMC12399589; doi:10.3389/fimmu.2025.1600250)
Supplement: Supplementary file 1 [file DataSheet1.docx]

Supplementary Table 1. Evaluation of Differential Diagnoses for Anemia in This Patient

| **Test** | **Result** | **Interpretation** |
| --- | --- | --- |
| Fecal occult blood test | Weakly positive | Prompted endoscopic evaluation |
| Gastroscopy | No bleeding source | Excluded upper GI bleeding |
| Colonoscopy | Normal findings | Excluded lower GI bleeding |
| LDH | 215 U/L (normal) | No evidence of hemolysis |
| Bilirubin (total/indirect) | 5.5/3.9 μmol/L (normal) | No hemolysis |
| Direct Coombs test | Negative | No autoimmune hemolysis |
| Vitamin B12 | 326.0 pmol/L | No nutritional deficiencies |
| folate | 11.80 nmol/L | No nutritional deficiencies |
| Serum ferritin | 271.6 ng/mL | Elevated – supports ACD |
| Transferrin saturation | 17.0 % | Suggests iron sequestration |
| Serum iron | 8.6 μmol/L (normal) | Not consistent with iron deficiency |

LDH：lactate dehydrogenase; ACD: anemia of chronic disease
